# Tethered Cord Syndrome After Myelomeningocele Repair: A Literature Update

**DOI:** 10.7759/cureus.10949

**Published:** 2020-10-14

**Authors:** Leopoldo Mandic Ferreira Furtado, José Aloysio Da Costa Val Filho, François Dantas, Camila Moura de Sousa

**Affiliations:** 1 Pediatric Neurosurgery, Vila da Serra Hospital, Nova Lima, BRA

**Keywords:** tethered cord syndrome, secondary tethered cord syndrome, myelomeningocele, fetal surgery

## Abstract

Tethered cord syndrome (TCS) after myelomeningocele (MMC) repair (or secondary TCS) is a challenging condition characterized by neurological, orthopedic, and urological symptoms, which are combined with a low-lying position of the conus medullaris and damage to the stretched spinal cord owing to metabolic and vascular derangements. It has been reported that this syndrome affects, on average, 30% of children with MMC. In this review, we revisit the historical aspects of secondary TCS and highlight the most important concepts of diagnosis, treatment, and outcomes for secondary TCS as well as the current research regarding the impact of fetal MMC repair in the incidence and management of TCS. In the future, the development of synthetic models of TCS could shorten the learning curve of pediatric neurosurgeons, and research into the cellular proapoptotic features and increased inflammation biomarkers associated with TCS will also improve the treatment of this condition and minimize retethering of the spinal cord.

## Introduction and background

Myelomeningocele (MMC) is an open neural tube defect that affects an average of three out of 10,000 live births, a statistic that is probably underestimated in developing countries; it is associated with high healthcare costs throughout a patient’s lifespan [1-4]. MMC is morphologically characterized by a placode, zona epitheliosa, and junctional zone and is most commonly localized in the lumbar region. A placode is a non-neurulated spinal cord, and gets this name due to the formation of a plaque instead of the cylindric appearance of the neurulated spinal cord, and can be distinguished from surrounding tissue by its red color. Placodes are surrounded by abnormal vascularized tissue, known as zona epitheliosa, which is separated from the normal skin by the junctional zone.

**Figure 1:**
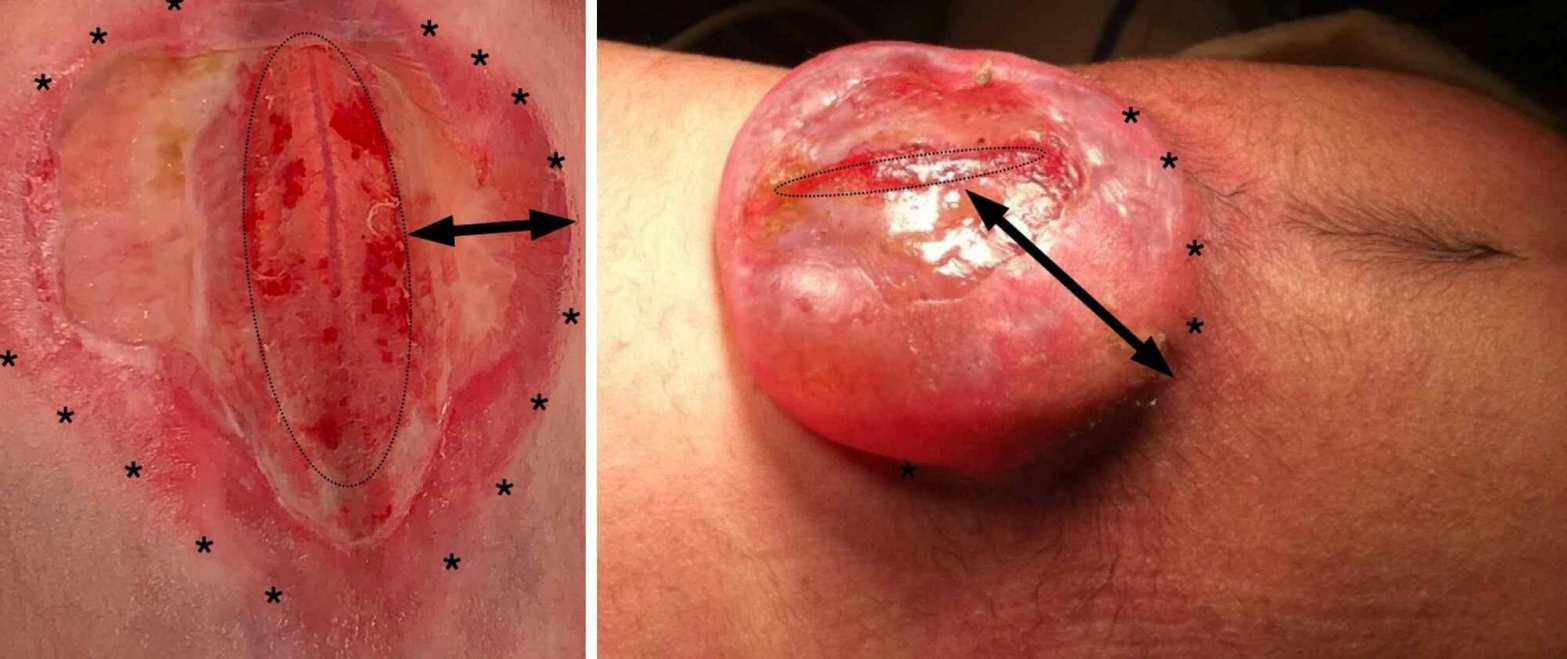
Anatomic landmarks of myelomeningocele Two different anatomic presentations of MMC are depicted: in a plane fashion (left) or a saccular fashion (right). In both presentations, the placode is perceived as a non-neurulated spinal cord, seen in the red ellipsoid structure (delimitated by elliptical dot-points). The zona epitheliosa is the abnormal and poor vascular tissue surrounding the placode (double-headed black arrows), and the junctional zone (*) is the delimitation point of the normal skin and the zona epitheliosa MMC: myelomeningocele

The classical MMC repair technique includes the separation of the placode from the zona epitheliosa, which is removed, and the placode is closed using a pial suture, promoting tunelization; this is followed by lateral exposition of the dura, which is sutured to covers the tunelized placode, and the normal skin is closed. At birth, the conus medullaris of MMC patients is below the normal anatomic level, usually at L1 or L2, which characterizes the anatomical tethered cord due to the attachment of the placode to the surrounding structures. During childhood growth, this attachment can lead to stretching of the spinal cord and becomes symptomatic in 30% of patients on average, who present with neurological, urological, and orthopedic issues [5]. This clinical condition is generally recognized as tethered cord syndrome (TCS) [6]. TCS is most commonly associated with thick filum terminale and other types of occult dysraphism as well as secondary scar formation after MMC repair [7]. Secondary TCS is the term used when the tethering occurs due to a primary surgical intervention, such as MMC. “Complex tethered cord” has been used synonymously with secondary TCS by Cochrane [8], Pierre-Kahn et al. [9], and Chapman [10], which highlights the anatomical challenges that surgical intervention represents.

The term “tethered spinal cord” was first coined in 1976 by Hoffman et al., who described 31 patients with elongated cords whose symptoms improved after sectioning of the filum terminale [11]. Nevertheless, other authors have also found an association between dysraphism and neurological symptoms [12,13]. Garceau coined the term “filum terminale syndrome” and reported a syndrome associated with a short, thick filum terminale, showing a short tau inversion recovery (STIR) signal. Furthermore, the surgical release of the abnormal filum resulted in the improvement of neurologic signs and the resolution of symptoms.

## Review

Pathophysiology

Experimental studies with animal models have highlighted meaningful mechanisms related to the increase in spinal function impairment observed in TCS. The main aspects that have been studied are metabolism, blood flow, and extensibility. Laboratory findings have shown that inflammation and proapoptotic substances also play a role.

Yamada et al. [6,14] performed an experimental study that determined the effect of tethering on the oxidative metabolism of cytochrome a/a3 using dual-wavelength reflection spectrophotometry and observed that untethering ameliorated the metabolism and that humans showed a similar reaction to untethering as cats. They considered some points of observation, such as the limitation of neurological deficits to the lumbosacral spinal cord due to the impairment of the oxidative metabolism found below the lowest pair of dentate ligaments (T12-L1). Nondermatomal sensory impairment associated with the gray matter is seriously affected by impaired oxidative metabolism. The conus medullaris displays the most affected oxidative metabolism impairment severity and the sphincteric outcome is also severe. Hypoxia has been identified as a factor of clinically relevant TCS, wherein the distraction of the spinal cord is thought to lead to vascular compromise with ischemic alterations occurring, especially in the gray matter [15]. Impaired movement of the spinal cord inside the spinal canal can also occur [16].

Previous studies have also elucidated the relationship between the elongation of the spinal cord under traction and the degree of metabolic dysfunction. In physiological conditions, the filum terminale, dentate ligaments, viscoelasticity of the spinal cord, and bulk of the lumbar spinal cord play a role in minimizing spinal cord injuries. According to concepts elucidated by experimental studies, the filum terminale and dentate ligaments work as buffers and, while the filum alleviates excessive elongation of the spinal cord by functioning as a rubber gun, the dentate ligaments give support to cervical, thoracic, and upper lumbar segments, counteracting the caudal traction force of the filum [7].

Another postulated mechanism has been attributed to the epipial layer of the spinal cord, which contains abundant collagen, and its extension narrows the cord and causes this layer to squeeze the interior of the cord, thus elevating intramedullary pressure with a “finger trap” effect. The cord becomes ischemic and metabolism ceases when the intramedullary pressure exceeds the perfusion pressure [17].

After MMC repair, features such as dysmorphic/misplaced placodio, shallow spinal canal, or absence of cerebrospinal fluid (CSF) around the placodio contribute to an increased probability of scar formation even when the surgeon has conducted correct dural closure [16,18,19].

In fact, in current studies, these events have been classified as cellular and molecular alterations in MMC placodes, and it has been found that they exhibited significantly elevated levels of glial fibrillary acidic protein and vimentin-immunoreactivity compared to normal spinal cords. Additionally, chemokines and cytokines, crucial mediators of secondary lesion cascades after spinal cord injury, were found to be elevated at the mRNA and immunoreactivity levels [20]. Cohrs et al. studied specimens collected from 12 patients who underwent untethering and identified specific pro-inflammatory and proapoptotic mediators that could underlie secondary TCS after MMC repair. They hypothesized that preventing these lesion cascades by applying anti-inflammatory and antiapoptotic factors, coupled with a meticulous surgical technique, could improve the retethering rate [21].

Epidemiology

Several studies have reported that the incidence of symptomatic TCS after MMC repair varies widely, ranging from less than 10% of patients up to 30% [12,16,22-24]. Regarding patients who have undergone fetal repair of MMC, statistically insignificant higher rates of tethering, as well as a higher incidence of dermoid cysts, have been reported [2]. This fact could be explained by the better functional activity of these patients, facilitating the recognition of tethering symptoms. Non-separation of transitional skin during the fetal repair could explain the incidence of cysts due to the incorporation of unrecognized young tissues during fetal surgery [2].

Usually, a relationship exists between an increase in symptoms and childhood growth spurts due to the lengthening of the spinal canal, which yields progressive spinal cord injuries related to the metabolic, vascular, and mechanical variables considered by previous experimental studies. The most common age to observe this rise in symptoms is represented by two peaks of incidence according to some reports: one is observed between two and four years and the other is observed between the ages of eight and 10 years; however, one study reports only one period, which occurs between the ages of five and nine years [16,25-28].

Clinical diagnosis

Although all patients have observable anatomical alterations of the spinal cord, TCS is fundamentally a clinical syndrome in which patients present with progressive neurological, orthopedic, and urological symptoms. These symptoms include worsening of ambulation due to leg weakness and club feet, impairment of bladder and bowel functions, recurrent urinary infections, and impaired sensory function. In addition, back or leg pain associated with dermatomal distribution and spasticity is also frequently reported [22,29,30]. In recent research that comprised a Danish population, in 45 out of 166 patients with MMC who underwent untethering, the most common indications were progressive spine deformity (40%), deteriorating ambulation (38%), and deteriorating neurogenic bladder and/or bowel dysfunction (32%) [31].

The possibility of shunt dysfunction mimicking symptoms of TCS can occur in MMC patients and the investigation of brain images is mandatory before the indication of spinal cord release. Sometimes, a shunt revision is indicated instead of untethering [16].

The assessment of patients by a multidisciplinary team consisting of orthopedists, physiotherapists, urologists, and neurosurgeons is paramount for the prompt recognition of TCS symptoms. Early diagnosis and treatment are essential to give patients the best possible chance of recovery. Furthermore, a delay in the diagnosis of secondary TCS is associated with irreversible injury [25,26,32].

Radiological diagnosis

**Figure 2:**
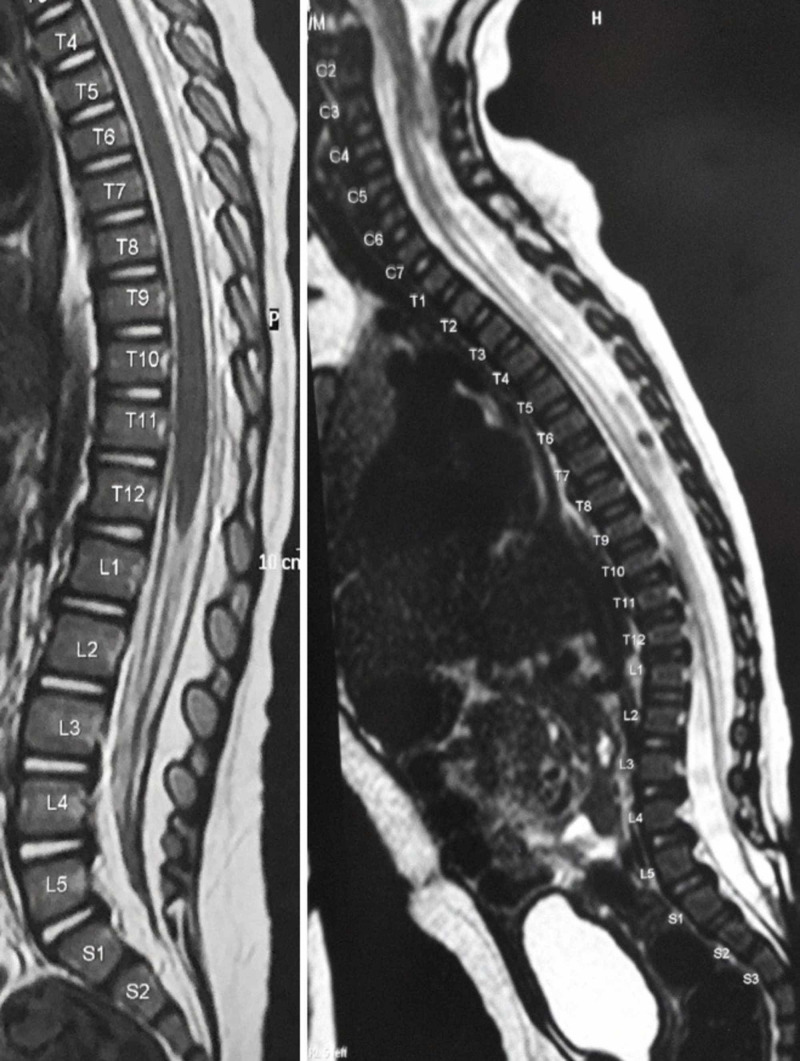
Normopositioning of conus medullaris and standard radiological findings of secondary tethered cord syndrome Normopositioning of the conus medullaris at the level of T12 on a sagittal T2-weighted spinal MR image (left) and low-lying position of the conus medullaris at S3 in a four-month-old child who underwent post-natal MMC repair and had no clinical symptoms of tethered cord syndrome (right) MMC: myelomeningocele

MRI is the main imaging procedure used to evaluate the anatomic features of a tethered spinal cord [33]. Several features, including hydromyelia, scoliosis, and spinal cord adhesions, are related to the severity of the tethering process. Although many patients who undergo MMC repair present with a low conus position after image investigation, this does not always imply a tethered cord [24,34] (Figure 2).

**Figure 3:**
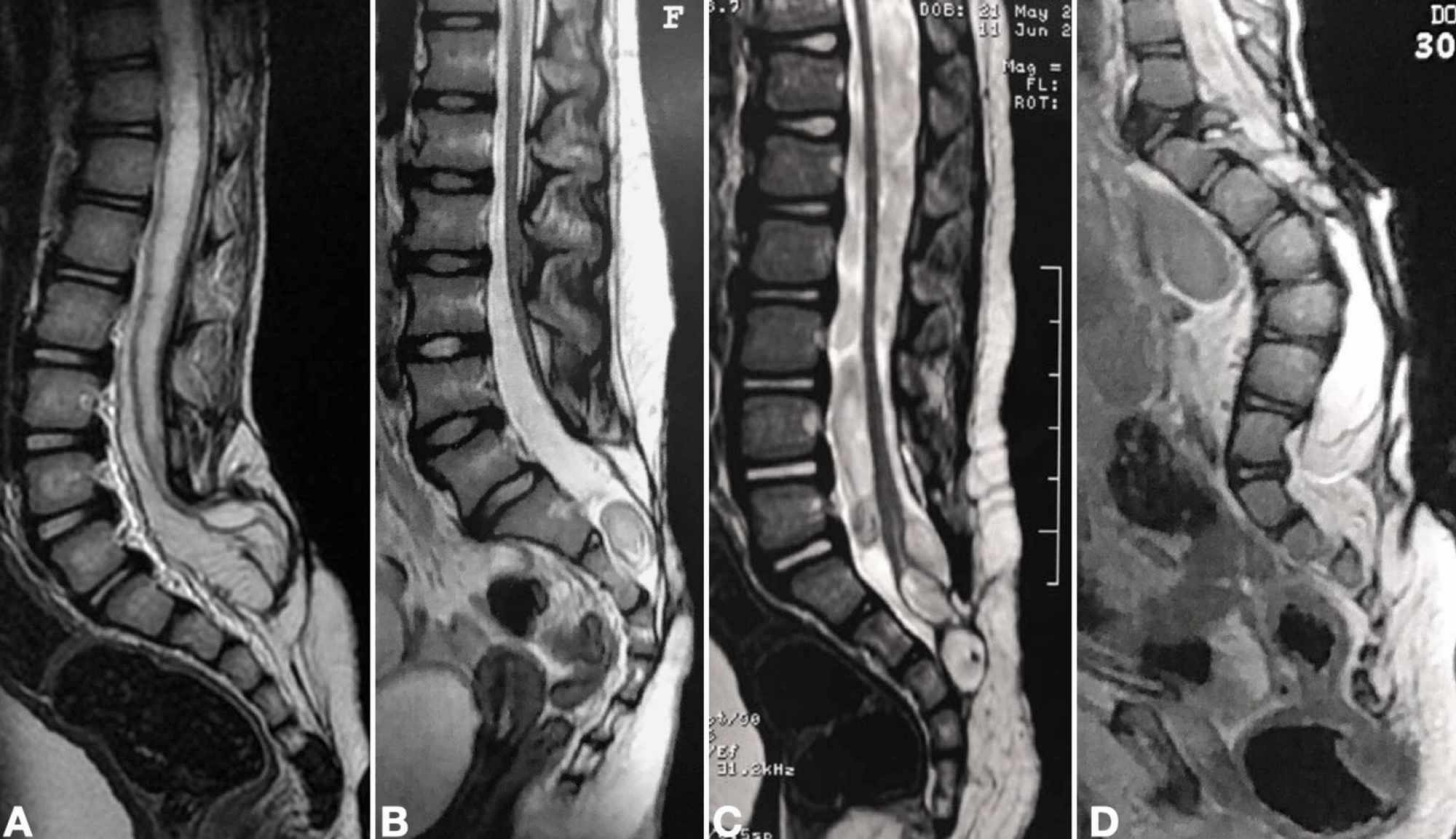
Features of several radiological tethering levels in children presenting with tethered cord syndrome after MMC repair Sagittal T2-weighted spinal cord MR image displaying severe syringomyelia with a large adherence of placode, classified as grade II (A). Patients in B and C presented as grade III. Low conus position was associated with a dermoid cyst in an MMC repaired postnatally (B) or exhibited two intramedullary epidermoid cysts in a child who underwent fetal repair of MMC (C). Lumbar kyphosis is associated with a low conus in grade IV (D) MMC: myelomeningocele

Taking this premise into account, Caldarelli et al. built a radiological scale based on the MRI imaging appearance of tethered cords and postulated four levels of tethering. Grade 1 implies a low-lying conus adherent to the lumbar scar, no evidence of adhesion along the spinal cord, and the conus medullaris is completely enveloped by the subarachnoid space. Grade II is featured by extensive adhesion of the placode and conus medullaris, with the placode incompletely enveloped by the subarachnoid space. In Grade III, there is abnormal tissue, such as lipoma or dermoid. Grade IV features severe kyphosis and/or scoliosis associated with features of Grade III [34]. Phase MRI of the longitudinal cord motions has offered some promise for predicting clinical tethering [35-37] (Figure 3).

The goals of untethering

Multiple studies have demonstrated the benefits of untethering [17,22,25-27,38]. The main goal of surgical intervention for TCS is to mechanically interrupt the excessive tension of the attached spinal cord in symptomatic children, in which there is compromised metabolism and circulation of the spinal cord owing to spinal cord stretching. However, surgery does not always reverse or even stabilize neurologic deterioration, and spinal cord surgery also carries a risk of incurring neurologic injury and impairment of function [14]. Additionally, interrupting contact between the spinal cord and the tethering pathology allows for the free flow of CSF with no adhesions to prevent retethering [25,35,39-41].

Strategies of surgical technique

Surgery planning should consider the anatomical aspects, such as the positioning of the placode, its relationship with the skin, and the complexity of scarring. Although MRI provides the best information, the use of CT fused with MRI is also a valid option [42]. The use of previous functional scales could also help understand and quantify the achievements of patients after surgery [34].

**Figure 4:**
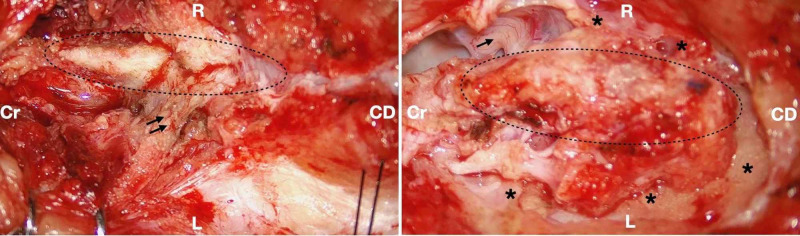
Microsurgical approach for tethered cord after MMC repair The pictures depict two steps of untethering. At the beginning of the microsurgical approach, the placode (area limited by an ellipse) is exposed and thick scar tissue (double black arrows) is seen dorsally (left). After circumferential untethering is performed, the placodium (ellipse) is released on both sides and caudally (*). The upper lumbar right nerve is seen (single black arrow) MMC: myelomeningocele; L: left side of the patient; R: right side of the patient; Cr: cranial; CD: caudal

Overall, untethering is challenging due to distorted anatomy and scarring from previous surgery. Scarring can contribute to an increase in the tension of neural structures at the site of surgical reconstruction (Figure 4) [15,21,25].

Ideally, the untethering should be approached from the upper level and should expose the normal cord in order to facilitate the progression of the dissection and recognition of the tethered region. Caution must be exercised to avoid damage to the placode attached to the scar tissue, and electrophysiological intraoperative monitoring should be conducted to determine the patient’s level of functionality and to minimize additional neurological injuries during surgery [16]. Furthermore, this procedure should release the placode in all directions, according to the grading system proposed by Kirollos and Van Hille, who considered three levels of untethering [43].

Refinements of the surgical technique, including the use of microscopic view to perform dural sutures over the placodio and dural substitutes, could minimize CSF fluid leak and retethering [19,40,44,45]. However, there is currently no technique that can reliably prevent the tethering of the spinal cord after MMC repair [18]. Recurrent tethering may occur because the spinal canal in babies is shallow and the neural contents are in direct contact with the posterior dura, facilitating adhesion [19].

The occurrence of multiple-repeat tethered cord release was defined by Maher et al. [46] in patients who underwent at least two subsequent tethered cord releases after primary MMC repair. Al-Holou et al. [27] performed a comparative study among patients who underwent first retethering and multiple retethering and found no differences in outcomes.

The role of electrophysiological intraoperative monitoring

The goal of electrophysical intraoperative monitoring is to minimize the risk of spinal cord injury during untethering procedures [25]. Pouratian et al. evaluated a retrospective cohort of patients who underwent untethering with the guidance of intraoperative monitoring and found that, across 46 procedures, decision-making during surgery was influenced by intraoperative neurophysiological findings in 41% of cases [25]. They recommend transection of the so-called autonomous placode, which they define as a placode that has compound muscle action potential when stimulated with low current (<10 mA) in its caudal segment and no potential in the upper segments, after which they observed improvements in spasticity and lower back pain.

Electrophysiological monitoring of TCS of patients who underwent fetal MMC repair showed increased potentials even below the anatomic level of MMC repair, suggesting good functional outcomes for this population [47].

Learning curve

During training, neurosurgery residents face difficulties when learning about the operative technique regarding untethering due to the great heterogenicity of cases. Furthermore, the commitment not to harm the functional spinal cord is a concern. Sometimes, the recognition of anatomy depends on the neurosurgeon’s experience.

The development of realistic simulations of congenital spine diseases is essential in order to shorten the learning curve of pediatric neurosurgeons. Efforts are currently underway to develop this kind of a model. Mattei et al. developed a synthetic model for pediatric spine pathologies, aiming to evaluate abilities such as recognition, duration of surgery, and the pressure used by the neurosurgeon [48].

Complications and postoperative care

Although the mortality rate associated with untethering is low, CSF leak followed by central nervous system infection is one of the most concerning side effects of untethering surgery and is reported in an average of 10-35% of cases [16]. This complication is associated with wound problems due to the absence of sufficient tissue to cover the placode and is linked to an increasing number of prior surgeries [46]. This could be addressed by planning with plastic surgeons in more complex cases. In our department, we recommend that the patient should remain flat for at least three days, and we use external lumbar drainage in order to minimize the risk of CSF leak and improve wound closure. In patients with CSF leakage, the initial treatment strategy is to maintain the patient in the plane decubitus position in order to minimize the CSF pressure under the skin as well as reinforcing sutures using sterile hydrocellular foam dressings. This kind of dressing precludes excessive manipulation and secondary infection of the skin. The reoperation is indicated only in cases where conservative measures have failed.

Outcomes

Concerning the main symptoms, such as pain, motor weakness, gait disturbances, scoliosis, and urinary disturbance, the majority of studies have reported an overall improvement or stabilization of symptoms, and increased importance has been attributed to an early diagnosis of TCS by a multidisciplinary team [25,26,49]. However, discrepancies in the percentage of overall improvement have been reported by some studies [22,38], and improvement of pain ranges from 75% of cases to 90% [26, 27]. This fact could be explained by the heterogenicity of patients regarding the dependency of variable previous sensory functions.

In a historical retrospective series, Al-Holou et al. considered the outcomes in the first six months and four years after spinal cord release. They applied functional scales and observed an improvement in back pain mainly in older patients. The worst outcomes were observed in patients who underwent prophylactic untethering for scoliotic fusion. Furthermore, motor function was shown to be improved in 7% of patients, and stabilization was seen in 88% of patients in the first six months. On extended follow-up, this rate changed to 68% for stabilization and 6% for gaining better motor function. They also found no statistical differences among patients who achieved complete circumferential untethering compared to those who did not [27].

Scoliosis is reported in 80% of secondary TCS patients. According to McLone et al., the relationship of cause and effect between TCS and scoliosis is indirect. They found that untethering stabilized or improved the curve in most children with curves under 50°, and they observed stabilization beyond one year in 63% of cases [50].

Herman et al. [26] reported on 153 patients who underwent secondary tethered cord release and 100 patients post-MMC repair. At an average of four years of follow-up postoperatively, motor discomfort improved by 63%, gait improved by 69%, and bladder function improved in 35% of cases. They attributed an important role to urology following urodynamic tests in the early diagnosis of bladder compromise to achieve better results for detethering.

## Conclusions

TCS is a clinical syndrome and its recognition must be prompt and early in order to increase the chances of better results. Patients who undergo fetal repair of MMC have a higher incidence of tethered cord symptoms and inclusion cysts, evidence that supports increased functionality below the level of anatomical closure. The best surgical approach to secondary TCS takes into account the features of spinal imaging and the use of intraoperative monitoring.

The development of synthetic models of TCS and research into cellular inflammation are some fronts of innovation to be improved for the treatment of tethered cord related to MMC.
